# Rapid Screening of MDR-TB in Cases of Extra Pulmonary Tuberculosis Using Geno Type MTBDR*plus*

**DOI:** 10.1371/journal.pone.0159651

**Published:** 2016-07-21

**Authors:** Richa Kumari, Rajneesh Tripathi, Alok Prakash Pandey, Tuhina Banerjee, Pallavi Sinha, Shampa Anupurba

**Affiliations:** Department of Microbiology, Institute of Medical Sciences, Banaras Hindu University, Varanasi, Uttar Pradesh, India; Hebrew University, ISRAEL

## Abstract

**Background:**

Drug resistance in tuberculosis is a major public health challenge in developing countries. The limited data available on drug resistance in extra pulmonary tuberculosis stimulated us to design our study on anti-tuberculosis drug resistance pattern in cases of extra pulmonary tuberculosis in a tertiary referral hospital of North India. We performed Geno Type MTBDR*plus* assay in comparison with conventional drug susceptibility testing by proportion method to study the mutation patterns in *rpoB*, *katG* and *inhA* genes.

**Methods:**

A total of 510 extra pulmonary samples were included in this study. After the smear microscopy, all the specimens were subjected for culture on Lowenstein Jensen (LJ) media. Phenotypic drug susceptibility testing (DST) was performed on LJ media for all the MTB isolates and compared with the results of Geno Type MTBDR*plus* assay which was performed with the DNA isolated from the culture by conventional method.

**Results:**

Of 510 specimens cultured, the total culture positivity obtained was 11.8% (60) encompassing 54 (10.6%) *Mycobacterium tuberculosis* and 6 (1.2%) non-tubercular mycobacteria (NTM). DST results by Geno Type MTBDR*plus* assay and solid culture methods were compared in 51 MTB isolates excluding the two *Rif* indeterminate and one invalid test. Geno Type MTBDR*plus* accurately identified 13 of 14 rifampicin-resistant strains, 14 of 15 isoniazid-resistant strains and 13 of 14 as multi drug resistant tuberculosis (MDR-TB) in comparison with conventional method. Sensitivity and specificity were 92.86% and 97.30% respectively for detection of RIF resistance, 93.33% and 94.44% respectively for detection of INH resistance, 92.86% and 97.30% respectively for detection of MDR-TB, while the overall concordance of Geno Type MTBDR*plus* assay with conventional DST was 94.11%. The turn-around time for performing Geno Type MTBDR*plus* assay test was 48 hours.

**Conclusion:**

The problem of MDR in extra pulmonary tuberculosis (EPTB) cannot be overlooked and due attention on patients should be given. Routine use of Geno Type MTBDR*plus* assay for the diagnosis of MDR-EPTB can substantially reduce the time between diagnosis and drug therapy. Culture along with Geno Type MTBDR*plus* assay could be a solution for rapid and accurate diagnosis of MDR-TB in low bacillary non sputum specimens.

## Introduction

Tuberculosis (TB), a major cause of morbidity and mortality, is the greatest killer worldwide alongside HIV due to a single infectious agent [1]. There were 6.0 million new TB cases in 2014 and 1.5 million TB deaths (1.1 million among HIV-negative people and 0.4 million among HIV-positive people), while an estimated 190 000 people died of MDR-TB in the year 2015 as reported by WHO [1]. Though Pulmonary TB is the most common presentation of the disease, extra pulmonary TB (EPTB) is also emerging as a serious clinical problem, accounting for 15–20 per cent of all the cases of tuberculosis and the percentage is much higher in HIV-positive patients, where it accounts for more than 50 per cent of all cases [2].

Drug resistance in tuberculosis is the major public health challenge globally. The first and foremost concern is to control drug resistance which makes the disease untreatable. Extra pulmonary tuberculosis (EPTB) refers to any bacteriologically confirmed or clinically diagnosed case of TB involving organs other than the lungs, e.g. pleura, lymph nodes, abdomen, genitourinary tract, skin, joints bones and meninges [3]. Though drug resistance in EPTB is not as common as in pulmonary tuberculosis yet the transmission of resistant strains is increasing the burden of multi drug resistant tuberculosis (MDR TB) even in extra pulmonary tuberculosis (EPTB). The early diagnosis of drug resistance is crucial to initiate appropriate therapy and avoid the devastating effect of MDR (multi drug resistance).

Emergence of MDR enforces an urgent need of a rapid method for determining antimicrobial susceptibility of isolates. The LJ culture based methods have been used historically for diagnosis and drug susceptibility testing from decades in resource limited countries. However MGIT recommended by World Health Organization and the U.S. Centers for Disease Control and Prevention can give results in a shorter period of time but requires special instrumentation and high cost[4], [5]. In this regard, Geno Type MTBDR*plus* assay, recommended by WHO for direct testing of sputum smear-positive specimens and on isolates of M. tuberculosis complex grown from smear-negative and smear-positive specimens, is a rapid method for diagnosis of MDR TB permitting detection of predominant mutations in genes *rpoB*, *katG*, and *inhA* [6]. There are very limited studies on Geno Type MTBDR*plus* assay in extra-pulmonary tuberculosis [7], [8].

Considering the fact that the paucibacillary nature of specimen in EPTB often leads to low sensitivity of AFB smear and culture, we proposed that culture along with molecular Geno Type MTBDR*plus* assay could be a better alternative for rapid identification of multi drug resistance in EPTB. Therefore this study was designed on comparison of Geno Type MTBDR*plus* assay and conventional drug susceptibility testing by proportion method for detection of MDR in extra pulmonary tuberculosis.

## Materials and Methods

### 2.1 Ethics statement

This study has been ethically approved by the Institute ethical committee of Institute of Medical Sciences (ECR/526/Inst/UP/2014), Banaras Hindu University, Varanasi. The given ethics committee waived the need for written consent since all the samples used were collected during the course of routine medical care based on clinicians’ request and further study was carried out on these samples which did not pose any additional risks to the patients.

### 2.2 Study design

This study was undertaken in the Department of Microbiology, Institute of Medical Sciences, Banaras Hindu University, Varanasi. Samples were collected from patients attending indoor and outdoor facility of Sir Sunderlal hospital for treatment during the period of August 2014 to July 2015. A provisional diagnosis of EPTB was made by the clinicians for all the patients included in this study based on the clinical symptoms. Patients included in the study presented with clinical symptoms like fever, night sweats, fatigue, loss of appetite, weight loss along with complaints specific to the body site. A total of 510 different extra pulmonary samples were analyzed during the said period. Single sample was collected from each patient.

### 2.3 Specimen collection and processing

All the samples were subjected to direct smear microscopy. Pus, gastric aspirate and other mucopurulent specimens were decontaminated by N-acetyl-L-cysteine and sodium hydroxide (NALC-NaOH) method, urine samples were concentrated by centrifugation prior to decontamination. The sediments were resuspended in 1–2 ml Phosphate-buffered saline (PBS) and inoculated on a pair of LJ media and one p-nitrobenzoic acid (PNB). PNB was used as a selective inhibitor of MTB. Body fluids from sterile sites, e.g. CSF, knee aspirate, pleural fluid, fine needle aspirates, bone marrow and ascitic fluid were inoculated directly without decontamination [9].

### 2.4 Identification of isolates

Isolates were first identified as *Mycobacterium tuberculosis* by their slow growth rate on Lowenstein Jensen (LJ) slants, colony morphology, sensitivity to p-nitrobenzoic (PNB) acid and further subjected to biochemical test (catalase test and nitrate reduction test). The isolates showing rapid growth on LJ along with resistance to PNB were examined microscopically and characterized as non tuberculous mycobacteria.

### 2.5 Drug susceptibility testing (DST)

Drug susceptibility test was performed by conventional 1% proportion method for all the isolates which were identified as *Mycobacterium tuberculosis*. The DST was carried out according to the guidelines of Revised National Tuberculosis Control Program (RNTCP) for first line anti tuberculosis drugs streptomycin, isoniazid, rifampicin and ethambutol with a concentration of 4μg/ml, 0.2μg/ml, 40μg/ml and 2μg/ml respectively [10].

### 2.6 Geno Type MTBDR*plus*

According to WHO recommendation Geno Type MTBDR*plus* was performed in three separate rooms [6]. Genotype MTBDR*plus* (Hain Lifescience GmbH) assay was performed according to the manufacturer's instructions. DNA extraction from the cultures of confirmed TB growth was done by CTAB-chloroform method with some modifications [11]. Multiplex PCR was performed using 45μl amplification mix consisting of 10μl AM- A and 35μl AM-B. 5μl DNA template was added in each tube in a separate room and amplification was performed with final volume of 50μl using a thermal cycler and amplification protocol provided by Hain Lifescience. Hybridization was performed in TwinCubator as per instructions provided by the manufacturers. After completion of hybridization, strips were washed, removed and fixed to GenoType MTBDR*plus* assay worksheet for interpretation [12]. Each strip of Geno Type MTBDR*plus* had 27 reaction zones (bands), including six controls (conjugate, amplification, *M*. *tuberculosis* complex (TUB), *rpoB*, *katG* and *inhA* controls), eight *rpoB* wild-type (WT1–WT8) and four mutant probes (*rpoB* MUT D516V, *rpoB* MUT H526Y, rpo*B* MUT H526D, and *rpoB* MUT S531L), one *katG* wild-type and two mutant probes (*katG* MUT S315T1 and *katG* MUT S315T2), and two *inhA* wild type and four mutant probes (*inhA* MUT1 C15T, *inhA* MUT2 A16G, *inhA* MUT3A T8C, *inhA* MUT3B T8A) [13]. Absence of wild-type band or the presence of mutant band was taken as an indication of a resistant strain. Incomplete amplification of RIF and/or INH genes or absence of TUB with an evaluable resistance pattern was considered as an invalid result and the test samples were repeated.

### 2.7 Statistical analysis

Data were analyzed by using an online diagnostic test evaluation tool, MedCalc. Sensitivity, specificity, negative predictive value, and positive predictive value with 95% confidence intervals were calculated. LJ culture and phenotypic resistance were considered gold standard for comparison.

## Results

A total of 510 samples from 510 patients, 318 males and 192 females, with a mean age of 24 (18, SD) years were evaluated in this study. The sample distribution was pus (304), gastric aspirate (91), pleural fluid (62), cerebrospinal fluid (18), urine (14), knee aspirate (4), fine needle aspirates (4), bone marrow (6), cold abscess (3), ascitic fluid (4). Among all the samples subjected to AFB, 49(9.60%) were found to be smear positive while 461 (90.39%) were smear negative. Of 49 smear positive samples, only 41 (83.67%) were able to grow on solid media. The total culture positivity obtained was 11.8% (60), while 84.3% (430) were culture negative and the remaining 3.9% (20) were contaminated. Of these 60 culture positive isolates, 54 (10.6%) showed growth on LJ only and 6 (1.2%) on both LJ and PNB, which were further characterized as *Mycobacterium tuberculosis* and non-tubercular mycobacteria (NTM) respectively.

### 3.1 Results of DST by Proportion method and Geno Type MTBDR*plus* assay

First line DST and Geno Type MTBDR*plus* assay was performed for all the 54 MTB isolates. The Geno Type MTBDR*plus* assay correctly identified *M*. *tuberculosis* in 53 of 54 *Mtb* culture positive samples with one invalid test. Of the culture positive specimens, two *Rif* indeterminate and one invalid Geno Type MTBDR*plus* test were excluded from the study. Phenotypic DST identified MDR-TB in 14/51 (27.45%), INH mono resistance in 1/51 (1.96%) and the remaining 36/51 (70.6%) were susceptible to both isoniazid and rifampicin. The sensitivity and specificity of GenoType MTBDR*plus* in detecting resistance to rifampicin, isoniazid and MDR-TB is detailed in Table 1.

**Table 1:** Performance of Geno Type MTBDR*plus* assay as compared to 1% proportion method in detecting resistance to rifampicin, isoniazid, and MDR-EPTB in 51 extra pulmonary clinical isolates.

			Isoniazid		
Genotype MTBDR*plus* assay			Phenotypic DST		
	**Resistant**	**Susceptible**	**Total**	**Sensitivity**	93.33
**Resistant**	14	2	16	**Specificity**	94.44
**Susceptible**	1	34	35	**NPV**	97.14
**Total**	15	36	51	**PPV**	87.50
			**Rifampicin**		
	**Resistant**	**Susceptible**	**Total**	**Sensitivity**	92.86
**Resistant**	13	1	14	**Specificity**	97.30
**Susceptible**	1	36	37	**NPV**	97.30
**Total**	14	37	51	**PPV**	92.86
			**MDR-EPTB**		
	**Resistant**	**Susceptible**	**Total**	**Sensitivity**	92.86
**Resistant**	13	1	14	**Specificity**	97.30
**Susceptible**	1	36	37	**NPV**	97.30
**Total**	14	37	51	**PPV**	92.86

MDR-EPTB = multi-drug resistant Extra pulmonary tuberculosis; NPV = negative predictive value; PPV = positive predictive value, CI = 95%

### 3.2 Concordance between Geno Type MTBDR*plus* assay and Proportion method

For assessing the performance of Geno Type MTBDR*plus* assay 1% proportion method was taken as gold standard. Geno Type MTBDR*plus* assay accurately identified 13 of 14 rifampicin-resistant-strains, 14 of 15 isoniazid-resistant strains and 13 of 14 as MDR-TB. Discordant result for detection of resistance to rifampicin, isoniazid, and MDR was obtained for one strain each. Genotypic resistance was detected in 3 samples (2 were INH resistant and 1 was MDR) which were sensitive by phenotypic testing (Table 2). The overall concordance between Geno Type MTBDR*plus* assay and phenotypic DST was 48/51 (94.11%).

**Table 2:** Concordance between Geno Type MTBDR*plus* assay and phenotypic DST.

Drugs	Concordant Results		Discordant results	
	Resistant by both methods	Sensitive by both methods	Sensitive by conventional DST but resistant by Geno Type MTBDR*plus* assay	Resistant by conventional DST but sensitive by Geno Type MTBDR*plus* assay
**Rifampicin**	13	36	1	1
**Isoniazid**	14	34	2	1
**MDR**	13	36	1	1

### 3.3 Band patterns in Geno Type MTBDR*plus* assay

**Fig 1:**
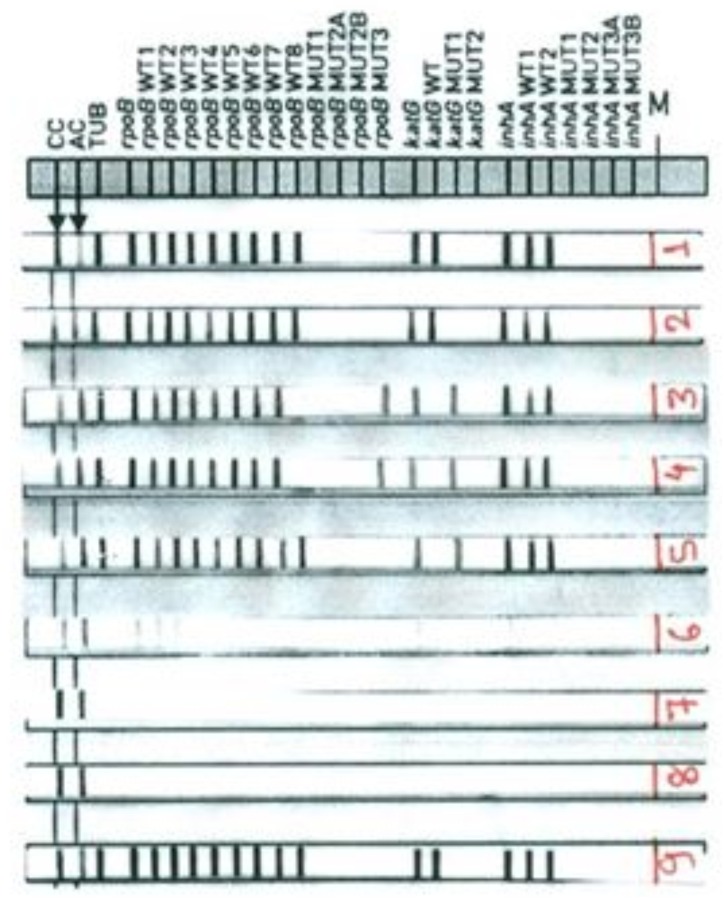
Band patterns of Geno Type MTBDR*plus* assay. **Lane 1 and lane 2:** Susceptible to rifampicin (RIF) and isoniazid (INH), **Lane 3 and lane 4:** MDR- TB (*rpoB* S531L mutation and *katG* S315T1 mutation), **Lane 5:** isoniazid monoresistant (*katG* S315T1 mutation), **Lane 6:** Absence of TUB band (invalid result), **Lane 7:** DNA negative control, **Lane 8:** Master mix negative control, **Lane 9:** Positive control (susceptible to rifampicin and isoniazid)

Out of 14 MDR-TB strains 12 had mutations in *rpoB* S531L (MUT3 band) and 2 had mutations in *rpoB* MUT D516V (MUT1 band). *katG* mutation was predominant in all isoniazid resistant strains. All 14 MDR-TB strains and one INH mono resistant strain had mutations in *katG* S315T1 (MUT1 band). Combined *KatG* and *inhA* mutations were found in one MDR-TB strain (Table 3). Representative band patterns obtained through Geno Type MTBDR*plus* assay are illustrated in Fig 1.

**Table 3:** Band patterns obtained through Geno Type MTBDR*plus* assay.

Gene	Band	Gene region/mutation	INH mono-resistant (n = 1)	MDR EPTB (n = 14)
rpoB				
	WT1	506–509		14(100)
	WT2	510–513		14(100)
	WT3	513–517		11(78.57)
	WT4	516–519		11(78.57)
	WT5	518–522		14(100)
	WT6	521–525		14(100)
	WT7	526–529		14(100)
	WT8	530–533		3(21.42)
	MUT1	D516V		3(21.42)
	MUT3	S531L		11(78.57)
katG				
	WT1	315	0	0
	MUT1	S315T1	1(100)	14(100)
inhA				
	WT1	-15/-16	1(100)	13(92.85)
	WT2	-8	1(100)	14(100)
	MUT1	C15T	0	1(7.14)

## Discussion

This study shows that the MTBDR*plus* assay may potentially contribute to shorten the time for detection of MDR-TB by offering a simple protocol that can be completed within 24 hours. Rapid, early and accurate diagnosis of tuberculosis improves outcomes and allows for timely intervention to prevent spread, especially in a high TB burden country like India which accounts for 27% of global TB notifications as reported by WHO[1]. Delayed and incorrect diagnosis impose extra load of drug administration, its side effect and is a key factor behind development of MDR- TB which ultimately leads to extensively drug resistant-tuberculosis (XDR-TB). Being a non- communicable disease, and its milder infectivity EPTB is not considered as a public health problem, of the same magnitude as that of its pulmonary form. But non-specific clinical presentation of disease and low bacillary load in specimens often make the diagnosis more difficult than pulmonary tuberculosis. Studies have suggested an excellent performance of MTBDR-Plus in comparison with MGIT liquid culture in paucibacillary samples of HIV infected patients. [14].

Based on the combination of multiplex polymerase chain reaction followed by reverse hybridization of amplicons to a strip with immobilized probes covering wild type and mutation sequences, Geno Type MTBDR*plus* assay proves to be a promising rapid diagnostic tool with lesser biohazard risk and short turn-around time. In this study with a shorter turnaround time of 24–48 hour Geno Type MTBDR*plus* assay showed a good concordance with conventional DST.

In our study, we found 10.6% culture positivity of *Mycobacterium tuberculosis* from 510 different extra pulmonary samples which is in agreement with the previous reports, where a positivity rate of 10.5% and 9.14% was reported [15,16]. A higher culture positivity of 30.1% was reported by Maurya *et al* [17]. This variation in positivity may well be due to geography and the use of liquid media. Forty one (83.67%) smear positive specimens were found to be culture positive in our study probably due to uneven distribution of bacilli in the sample or non-viable bacilli. Though very few data is available on drug resistance in extra pulmonary tuberculosis, previous studies have reported 12.5% MDR- EPTB in Nepal and 10% in Delhi India [15], [16]. We got a higher prevalence of 27.45% MDR-EPTB in our study.

Sensitivity and specificity of 93.33% and 94.44% for the detection of isoniazid resistance is concordant with the studies where a comparable sensitivity of 93% and specificity of 97% were seen [18]. The specificity of 97.30% in case of rifampicin resistance is in agreement with the report of Huyen *et al* [19] and the sensitivity of 92.86% in our study was within the range of 92 to 99% of previous reports [20],[21],[22]. The sensitivity and specificity of Geno Type MTBDR*plus* assay for MDR-TB detection in our study was 92.86% and 97.30% which is comparable to the reports, where 92.3% and 96.2% of sensitivity and specificity was reported [23].

The commonest mutation associated with rifampicin resistance lies in 81 base pair region (codon 527 to 533) of the *rpoB* gene [24]. In our study mutation in codon s531L was detected in 78.57% of RIF resistant isolates, which is in corroboration with other studies [22]. The 100% association of mutation in codon s315T1 of katG with INH resistance in this study is in affirmation with earlier reports [25]. However several reports have shown a lower prevalence of katG mutation [19]. Combined mutation in inhA and katG gene was 7.4% which is within a range of previously reported studies [10].

Another molecular diagnostic method introduced by WHO is Xpert MTB/RIF which is based on real time PCR. Processing directly from samples is a major advantage of this technique over the MDRTB*plus* assay performed on a positive culture isolate. Despite being a rapid technique with a turn-around time of 3 hours for detection, this technique can detect rifampicin resistance only. The reports have shown a poor sensitivity of this technique, furthermore they have suggested a strong need to evaluate the performance of the Xpert MTB/RIF due to its high false negative results [26]. There is a need of a more thorough evaluation of Xpert in EPTB.

Our study strongly acclaims the routine use of Geno Type MTBDR*plus* assay for the diagnosis of MDR-EPTB which can substantially reduce the time between diagnosis and drug therapy. The shorter turn-around time of 48 hours is a major advantage concomitant with this molecular test which gives it an edge over conventional DST, where the total turn-around time is 70 days comprising of 28 days for culture isolation and 42 days for DST.

Low rate of culture positivity is a limitation of this study. Liquid culture could have been done to increase the culture positivity where the yield is higher, especially in smear negative specimens [27]. Due to lack of clinical history of the patients in this study, we could not correlate the prevalence of drug resistance in newer or previously treated cases and other probable reasons for predisposition of multi drug resistance. Direct use of Geno Type MTBDR*plus* assay in extra-pulmonary specimens could have further reduced the time for detection of MDR-TB from several weeks to few days [8]. Study on a bigger sample size and including the data from histo pathological examination of each patient could have added more value to this study. There is a need to consider the impact of detecting genotypic resistance in patients who don’t have phenotypic resistance. They will be started on more toxic MDR TB treatment. Hence identification of the nature of mutation is needed for accurate diagnosis and treatment of drug resistance [28].

From the present study it is concluded that the problem of MDR in extra pulmonary tuberculosis cannot be overlooked. Our study strongly recommends the use of culture along with MTBDR*plus* assay for the diagnosis of drug resistance in extra pulmonary tuberculosis. Despite some limitations like need for an appropriate infrastructure, trained and skilled laboratory personnel, Geno Type MTBDR*plus* assay has proven to be a highly sensitive, specific and rapid diagnostic technique.

This requires validation with larger number of samples and more studies on drug resistance in extra pulmonary tuberculosis.
